# Neuropsychological outcome after carbon monoxide exposure following a storm: a case-control study

**DOI:** 10.1186/1471-2377-14-153

**Published:** 2014-07-21

**Authors:** Bérengère Pages, Mélanie Planton, Sophie Buys, Béatrice Lemesle, Philippe Birmes, Emmanuel Joseph Barbeau, Stéphanie Maziero, Laurie Cordier, Claudine Cabot, Michèle Puel, Michèle Genestal, François Chollet, Jérémie Pariente

**Affiliations:** 1 Service de Neurologie, Pôle Neurosciences, Centre Hospitalier Universitaire de Toulouse, CHU Purpan, Place du Dr Baylac, Toulouse Cedex 9, France; 2 Inserm, Imagerie Cérébrale et Handicaps Neurologiques UMR 825, Université de Toulouse, UPS, CHU Purpan, Place du Dr Baylac, F-31059 Toulouse Cedex 9, France; 3 Service de Réanimation, Centre Hospitalier Universitaire de Toulouse, CHU Purpan, Place du Dr Baylac, Toulouse Cedex 9, France; 4 Université de Toulouse; UPS; Laboratoire du Stress Traumatique (LST-JE 2511), Centre Hospitalier Universitaire de Toulouse, CHU Purpan, Place du Dr Baylac, Toulouse Cedex 9, France; 5 Centre de Recherche Cerveau et Cognition (CERCO), Pavillon Baudot Centre Hospitalier Universitaire de Toulouse, CHU Purpan, Place du Dr Baylac, Toulouse Cedex 9, France; 6 Centre Antipoison et de Toxicovigilance, Centre Hospitalier Universitaire de Toulouse, CHU Purpan, Place du Dr Baylac, Toulouse Cedex 9, France

**Keywords:** Carbon monoxide, Neuropsychological outcome, Storm, Memory, Post-traumatic stress disorder

## Abstract

**Background:**

The cognitive consequences of carbon monoxide (CO) poisoning are well described. However, most studies have been carried out without an ad-hoc group of control subjects. The main aim of this study was to evaluate cognitive and psychiatric outcome after CO exposure during the storm Klaus in the South West of France (January 2009) in a homogeneous group of patients compared to a group of 1:1 paired controls.

**Methods:**

Patients and controls were asked to fill out questionnaires about quality of life and cognitive complaints. They then underwent a cognitive assessment derived from the Carbon Monoxide Neuropsychological Screening Battery. Psychiatric assessment was performed using subtests of the Mini International Neuropsychiatric Interview.

**Results:**

38 patients and 38 paired controls were included (mean age 38.8 years) and evaluated 51 days after the poisoning. No difference was found between groups on the cognitive complaint questionnaire but patients had a lower quality of life than controls. Patients showed significantly lower cognitive performance than controls on processing speed, mental flexibility, inhibition and working and verbal episodic memories. Patients were more depressed than controls, and suffered more from post-traumatic stress disorder.

**Conclusions:**

We report the first study investigating cognitive and psychiatric outcome in consecutive patients after CO poisoning during a natural disaster, using a group comparison method. CO poisoning during storms needs to be dealt with adequately and clinicians should be aware of its possible consequences.

## Background

The cognitive consequences of carbon monoxide (CO) poisoning are well described [1]. They can be observed during the acute phase of the poisoning, after a few days and persist for over a year [2-6]. They mainly involve memory, attention, processing speed and executive functions [6]. However, most studies have been carried out without an ad-hoc group of control subjects, mainly relying on published norms for comparison [7,8]. Interestingly, Deschamps et al. did not evidence any difference between patients and controls when a specific group of controls was recruited [9]. These last studies gathered patients with variable circumstances of CO intoxication, which may be the reason for their heterogeneous results, but which also questions the extent to which results apply to all patients. It was to avoid these limitations that we carried out the present study in a homogenous group of intoxicated patients using rigorously selected control subjects.

The storm Klaus reached the South West of France between 23^rd^ and 25^th^ January 2009 leaving 1,745,000 households without electricity. It is considered to be the most violent storm in France in the past decade, resulting in 1.2 billion Euros of structural damage. Alternative means of heating and lighting, including electricity generators, were used and resulted in increased CO poisoning. 117 persons were poisoned in the Midi-Pyrenean region and referenced by the local specialized center.

The main aim of this study was to evaluate cognitive and psychiatric outcome after CO exposure during the Klaus storm in a homogeneous group of patients and to compare the results with those obtained in a group of controls paired 1:1 to patients for age, gender and education level.

## Methods

### Subjects

We contacted 117 poisoned patients by phone to invite them to participate in the present study. We invited them to attend an outpatient clinic for a cognitive and psychiatric assessment in the Purpan hospital, Toulouse, France.

Patients were eligible for the study if they met the following criteria: 1) A diagnosis of CO poisoning according to the published criteria of documented exposure to carbon monoxide or obvious exposure to carbon monoxide with observation of any of the following symptoms: loss of consciousness, confusion, headache, malaise, fatigue, forgetfulness, dizziness, visual disturbances, nausea, vomiting, cardiac ischemia, or metabolic acidosis [6]. If the carboxyhemoglobin level was below 10 percent, the patient was eligible only if carbon monoxide poisoning was the only plausible diagnosis, 2) Above 15 years of age, 3) French language abilities good enough to undergo the assessment, 4) Signed informed consent. Non-inclusion criteria were: patients admitted to a nursing home, patients with a preexisting chronic neurological illness or with depression or post-traumatic stress disorder, patients with a life threatening condition and patients with hypoxia due to a chemical intoxication. Patients who suffered from any medical condition, other intoxication or brain traumatism between CO poisoning and the assessment in the present study were not included. When possible, carboxyhemoglobin (COHb) level was recorded. A CT scan was performed when necessary at the acute phase.

### Procedure

The included patients were asked to fill out cognitive complaint and quality of life questionnaires [10,11]. They then underwent general and neurological clinical examinations, which included a semi-structured interview. A cognitive assessment derived from the Carbon Monoxide Neuropsychological Screening Battery was performed: Free and Cued Selective Reminding Test (FCSRT) for verbal episodic memory, WAIS-III Letter-Number Sequencing for working memory, MEM-III orientation test, WAIS-III Digit Symbol Test, TMT A and B, Stroop test for executive functions, and confrontation naming test for language [4]. In accordance with the literature, 8 specific variables of interest were identified within this battery: the cued and total 3 recall of the FCSRT (score/48), the raw score for Letter-Number Sequencing (/21), the raw score of the orientation test (/14), the raw score for digit substitution in the Digit Symbol Test (/133), time in seconds for TMT B-A, reading time in seconds for the score interference part of the Stroop test and the raw score for naming in the confrontation naming test (/10) [6-8]. Psychiatric assessment was performed using subtests of the Mini International Neuropsychiatric Interview (M.I.N.I. 5.0.0): major depressive episode (MDE), manic episode, hypomanic episode, post-traumatic stress disorder (PTSD), psychotic disorders, and antisocial personality disorder [12]. The total time of the evaluation was approximately 1 hour.

A group of controls not exposed to carbon monoxide was enrolled in the study. They were relatives of patients seen in our memory clinic. They were paired 1:1 to patients for age, gender, and level of education. Controls received exactly the same evaluation tests as patients.

Patients and controls gave their informed consent for this study. The study was approved by the local ethics committee (“Comité d’Ethique de la Recherche” of the Toulouse teaching hospital “CHU de Toulouse”, France).

### Statistical analysis

We performed an intergroup comparison on demographic and clinical data using bilateral Student t tests for independent samples, non-parametric Mann-Whitney U tests when a Kolmogorov-Smirnov test indicated that the sample did not follow a normal distribution, or a chi^2^ test when appropriate. Effect size was estimated using Cohen’s D [13] when a significant difference was observed. Following conventional criteria, an effect size of 0.20 to 0.30 was considered “small”, around 0.50 “medium” and above 0.80 “large”. In the patients’ group, we used Spearman correlation between COHb level and cognitive composite score. The cognitive composite score was generated from the 8 specific variables of interest previously identified (the cued and total 3 recall of the FCSRT, the raw score for Letter-Number Sequencing, the raw score for the orientation test, the raw score for digit substitution in the Digit Symbol Test, time in seconds for TMT B-A, reading time in seconds for the score interference part of the Stroop test and the raw score for naming in the confrontation naming test). For each of the 8 variables, the patients’ scores were standardized according to the group average of the variable. Then, the cognitive composite score was calculated as the mean of the 8 standardized cognitive scores. The lower the cognitive composite score, the more severe the cognitive impairment. *p* < 0.05 was considered statistically significant.

## Results

**Figure 1:**
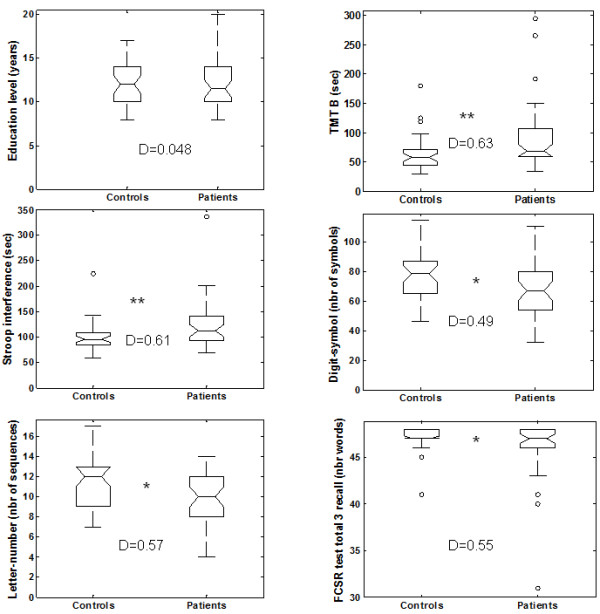
**Group comparisons for education level and the five cognitive tests that showed significant differences.** For the main measure of each test, we provide a graphical representation of the dispersion of the performance of each group using box-plots. Boxes represent the 25^th^ and 75^th^ percentiles, and the lines in the boxes indicate the medians. Notches display the variability of the median between samples. Upper and lower lines of whiskers represent minimum and maximum performance. Circles are outliers in each group, i.e. subjects whose performance fell outside minimum or maximum values of +/- 1.5 the difference between the 25^th^ and 75^th^ percentiles. D represents Cohen’s D value. *indicates a significant difference (*: *p* < 0.05 and **: *p* < 0.01).

Among the 117 patients, 30 were children under 15 who were not contacted. 87 patients were contacted by phone between February 25^th^ and 27^th^ 2009. 7 did not meet the inclusion criteria. The 80 remaining persons were contacted and invited to participate. 42 persons did not want to enter the study and explained they had resumed their life with no sequelae. 38 persons were included (mean age 38.9 ± 16.6; 34.2% male; 11.7 ± 2.9 years of education). 38 paired controls were also recruited (mean age 38.7 ± 16.5; 34.2 % male; 11.4 ± 3.9 years of education). No difference was found between patients and controls for age, gender and education level (Figure 1, data reported for education). Patients were assessed for the study 50.9 ± 17.3 days after CO poisoning. They all fulfilled the CO poisoning diagnosis criteria. The COHb level was recorded for 24 out of the 38 patients (10.9% (±7.9)). No CT scan lesion was observed. 2 patients (5%) had received hyperbaric oxygen therapy, 6 (16%) had had normobaric oxygen therapy and 29 (76%) had received both. For 1 patient, the treatment received was not known. Causes of CO intoxication, initial symptoms and symptoms reported during the semi-structured interview are reported in Table 1.

**Table 1:** Causes of CO poisoning, initial symptoms and symptoms observed during the study

**Cause of CO poisoning**	
Electric generator	58%
Gas heater	29%
Charcoal heater	8%
Gas water heater	5%
Initial symptoms*	
Headache	82%
Dizziness	55%
Nausea	53%
Fatigue	29%
Loss of consciousness	29%
Cardiac ischemia	10%
Severe ketoacidosis	3%
Acute pulmonary edema	3%
**Symptoms during study evaluation**	
Headache	47%
Fatigue	45%
Feeling sad	39%
Concentration difficulties	39%
Feeling apathetic	32%
Experiencing reviviscences	32%
Memory complaint	26%
Reduction of daily activities	26%
Still on sick leave	24%

*No patient presented paralysis, ventricular arrhythmia, convulsion, shock, coma, stroke or rhabdomyolysis as initial symptoms. No brain lesions assessed by CT scan was reported.

No difference was found between groups on the cognitive complaint questionnaire but patients had a lower quality of life than controls (patients: 103.8 ± 18.3; controls: 113.4 ± 9.2; *p* < 0.001; Table 2). Patients showed significantly lower cognitive performance than controls on 5/8 of the following cognitive variables of interest: FCSRT, letter-number sequences, digit symbol test, TMT B test, Stroop test (Figure 1). The effect size was “medium” for all these tests (Figure 1). Different results between the two groups were identified in two of the six subtests of the M.I.N.I.: major depressive disorder (*p* < 0.01) and PTSD (*p* < 0.02) were more common in the patient group (Table 2).

**Table 2:** Patients’ and controls’ cognitive, psychiatric and global scores

	**Patients**	**Controls**	***p *** **value**	**Cohen’s D**
	**(n = 38)**	**(n = 38)**		
**Cognitive assessment**				
*Disorientation in time and space (MEM III)*				
MEM III, orientation (/14)	13.76 (±0.48)	13.86 (±0.34)	0.281	-
**Memory**				
*Free recall*				
FCRST (sum of 3 free recall, /48)	11.21 (±2.30)	11.86 (±2.32)	**0.048**	0.47
FCRST (delayed free recall, /16)	13 (±2.02)	13.92 (±1.51)	**0.028**	0.52
*Cued recall*				
FCSRT (sum of 3 total recall, /48)	14.63 (±1.66)	15.36 (±0.88)	**0.030**	0.55
FCSRT (delayed total recall, /16)	15.63 (±0.91)	15.92 (±0.35)	0.073	-
**Executive functions**				
*Processing speed*				
TMT A time	36.73 (±17.64)	31.13 (±12.04)	0.110	-
Stroop test (denomination time)	64.21 (±14.58)	59.05 (±8.39)	0.063	-
Stroop test (reading time)	48.31 (±10.07)	41.71 (±6.54)	**0.001**	0.79
Digit symbol test (/133)	67.92 (±17.80)	76.71 (±17.74)	**0.034**	0.49
*Working memory*				
Letter-Number sequencing (WAIS III, /21)	9.97 (±2.56)	11.36 (±2.36)	**0.016**	0.57
*Flexibility*				
TMTB time	36.73 (±17.64)	31.13 (±12.04)	**0.010**	0.63
TMT B-A time	54.55 (±43.57)	32.24 (±24.13)	**0.008**	0.64
TMT B-A Errors	0.08 (±0.68)	0.13 (±0.34)	0.685	-
*Inhibition*				
Stroop test (interference score, time)	59.65 (±38.36)	39.86 (±21.37)	**0.007**	0.61
Stroop test (interference score, non-corrected errors)	0.47 (±1.51)	0.02 (±0.16)	**0.013**	0.59
**Language**				
Denomination (/10)	9.95(±0.23)	9.95 (±0.23)	1.000	-
**Psychiatric assessment**				
MNI, major depressive episode, n (%)	8 (21.05%)	0	**0.003**	-
MNI, manic episode, n (%)	2 (5.26%)	1 (2.63%)	0.556	-
MNI, hypomanic episode, n (%)	2 (5.26%)	0	0.152	-
MNI, post-traumatic stress disorder, n (%)	6 (15.78%)	0	**0.011**	-
MNI, psychotic disorders, n (%)	1 (2.63%)	0	0.314	-
MNI, antisocial personality disorder, n (%)	0	0	-	-
**Global assessment**				
*Cognitive complaints*				
Mc Nair score (/45)	11.56 (±7.97)	13.52 (±5.00)	0.206	-
*Quality of life*				
MOS SF-36, Total (/135)	103.81 (±18.37)	113.42 (±9.29)	**0.020**	0.69

MEM III, Wechsler Memory Scale; FCRST, Free and Cued Selective Reminding Test ; WAIS III, Wechsler Adult Intelligence Scale; TMT, Trail Making Test; MINI, Mini International Neuropsychiatric Interview; MOS SF-36, Medical outcome Study Short Form 36.

**Figure 2:**
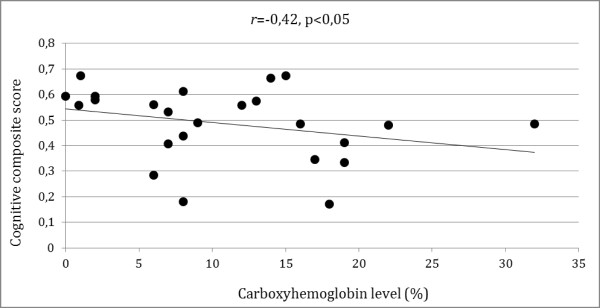
Spearman correlation between COHb level and cognitive composite score.

We found a negative correlation between patients’ COHb level and cognitive composite score (*r* = -0,42, *p* < 0,005) (Figure 2).

## Discussion

In this controlled study, patients were all poisoned during the same weather event and over a very short period of time (3 days). They were enrolled and assessed about 7 weeks after the intoxication. In this homogeneous group of patients, we evidenced both cognitive and psychiatric sequelae after CO poisoning.

Performance was significantly lower in patients than in controls in five out of the eight cognitive variables of interest, despite the fact that controls and patients had been rigorously matched. Three of the variables assessed processing speed and executive functions, one working memory and one verbal episodic memory. These results suggest that these patients were suffering from multi-domain cognitive impairment. We are aware of only two studies in which a group of patients was compared to a group of matched controls specifically enrolled for the study [9,2]. Surprisingly, in Deschamps’ study no cognitive difference was reported between the two groups but not all the patients underwent cognitive assessment [9]. Significant differences were showed in Chen’s study but neuropsychological assessment was only partial and memory function had not been assessed [2]. Other studies investigating cognitive outcome after CO poisoning did not specifically enroll a group of control subjects but reported results that are in accordance with ours [5,7,14,15]. We acknowledge that our study is cross-sectional. Therefore we did not address the delayed neuropsychological impairment issue. The cause of intoxication may be the reason for this apparent difference as previous studies have often included patients who sustained voluntary CO poisoning during suicide attempts, which is a more severe exposure than the involuntary intoxication that happened in our sample.

Psychiatric sequelae such as anxiety and depression have only been reported in a few studies [8,14]. Patients in our sample also reported PTSD after CO poisoning. This is congruent with the patients’ complaints about their reviviscences.

It is possible that the recruitment method was a factor of bias. The 42 intoxicated persons who did not enter the study might have had lower cognitive impairment compared to the 38 recruited intoxicated participants. Therefore, the size of the observed effects in our analysis is possibly overestimated. However, we have shown that cognitive complaint was as low in the patient group as it was in the group of controls. We acknowledge that if the recruitment method was not ideal, its influence on the results was probably weak.

## Conclusions

We report here the first study investigating cognitive and psychiatric outcome in consecutive patients after CO poisoning during a natural disaster. Using a group comparison method, we confirmed the potential multi-domain cognitive nature of sequelae PTSD is one aspect that is also to be taken into account after that specific intoxication. In spite of the medium size effect reported in this study, patients as a group reported a lower quality of life, which may have been caused by these cognitive and psychiatric sequelae. CO poisoning during storms must therefore be dealt with adequately and clinicians should be aware of its possible consequences.

## Competing interests

The authors declare that they have no competing interests.

## Authors’ contributions

BP participated in the design of the study, assessed patients’ neuropsychological functions and performed analysis and interpretation of data. MPlanton performed the statistical analysis and helped draft the manuscript. SB assessed patients’ neuropsychological functions and performed analysis and interpretation of data. BL participated in the design of the study and helped assess patients’ cognitive functions. PB met all subjects and performed their psychiatric assessment. EJB participated in the design of the study and analysis of data and was involved in drafting the manuscript. SM recruited and assessed control subjects’ neuropsychological functions. LC assessed patients’ neuropsychological functions and performed analysis and interpretation of data. CC participated in the design of the study. MPuel performed neurological assessment and participated in the design of study. MG participated in the design of the study. FC performed neurological assessment and participated in the design of study. JP conceived the study, performed neurological assessment and was involved in drafting the manuscript. All authors read and approved the final manuscript.

## Pre-publication history

The pre-publication history for this paper can be accessed here:

http://www.biomedcentral.com/1471-2377/14/153/prepub
